# Monoclinic polymorph of poly[aqua(μ_4_-hydrogen tartrato)sodium]

**DOI:** 10.1107/S1600536810000681

**Published:** 2010-01-09

**Authors:** Mohammad T. M. Al-Dajani, Hassan H. Abdallah, Nornisah Mohamed, Ching Kheng Quah, Hoong-Kun Fun

**Affiliations:** a School of Pharmaceutical Sciences, Universiti Sains Malaysia, 11800 USM, Penang, Malaysia; b School of Chemical Sciences, Universiti Sains Malaysia, 11800 USM, Penang, Malaysia; c X-ray Crystallography Unit, School of Physics, Universiti Sains Malaysia, 11800 USM, Penang, Malaysia

## Abstract

A monoclinic polymorph of the title compound, [Na(C_4_H_5_O_6_)(H_2_O)]_*n*_, is reported and complements an ortho­rhom­bic form [Kubozono, Hirano, Nagasawa, Maeda & Kashino (1993 ▶). *Bull. Chem. Soc. Jpn*, **66**, 2166–2173]. The asymmetric unit contains a hydrogen tartrate anion, an Na^+^ cation and a water mol­ecule. The Na^+^ ion is surrounded by seven O atoms derived from one independent and three symmetry-related hydrogen tartrate anions, and a water mol­ecule, forming a distorted penta­gonal–bipyramidal geometry. Independent units are linked *via* a pair of inter­molecular bifurcated O—H⋯O acceptor bonds, generating an *R*_2_^1^(6) ring motif to form polymeric two-dimensional arrays parallel to the (100) plane. In the crystal packing, the arrays are linked by adjacent ring motifs, together with additional inter­molecular O—H⋯O inter­actions, into a three-dimensional network.

## Related literature

For the optical activity of tartaric acid, see: Synoradzki *et al.* (2008 ▶). For Na—O distances, see: Wong *et al.* (2009 ▶). For the ortho­rhom­bic polymorph of C_4_H_5_O_6_Na·H_2_O, see: Kubozono *et al.* (1993 ▶). For hydrogen-bond motifs, see: Bernstein *et al.* (1995 ▶). For the stability of the temperature controller used for the data collection, see: Cosier & Glazer (1986 ▶).
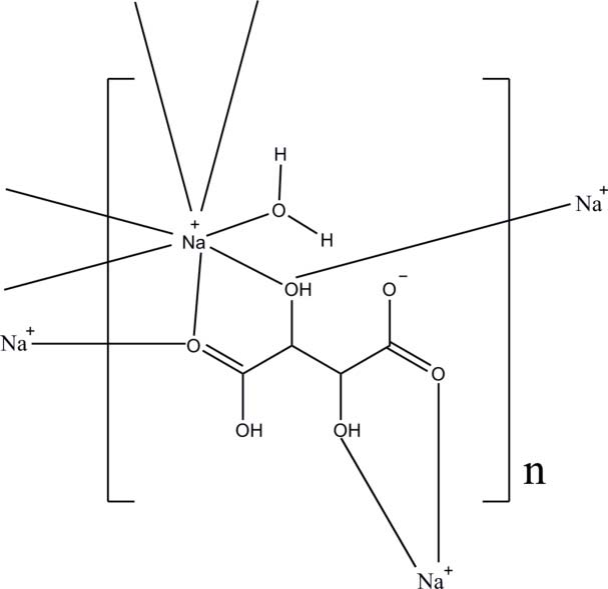


## Experimental

### 

#### Crystal data

[Na(C_4_H_5_O_6_)(H_2_O)]
*M* *_r_* = 190.09
Monoclinic, 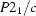

*a* = 8.9723 (2) Å
*b* = 7.1457 (1) Å
*c* = 12.0186 (2) Å
β = 119.571 (1)°
*V* = 670.18 (2) Å^3^
*Z* = 4
Mo *K*α radiation
μ = 0.24 mm^−1^
*T* = 100 K
0.40 × 0.09 × 0.05 mm

#### Data collection

Bruker SMART APEXII CCD area-detector diffractometer
Absorption correction: multi-scan (*SADABS*; Bruker, 2009 ▶) *T*_min_ = 0.895, *T*_max_ = 0.979
6649 measured reflections
1947 independent reflections
1532 reflections with *I* > 2σ(*I*)
*R*_int_ = 0.034

#### Refinement

*R*[*F* ^2^ > 2σ(*F* ^2^)] = 0.037
*wR*(*F* ^2^) = 0.092
*S* = 1.03
1947 reflections
109 parameters
H-atom parameters constrained
Δρ_max_ = 0.51 e Å^−3^
Δρ_min_ = −0.29 e Å^−3^

### 

Data collection: *APEX2* (Bruker, 2009 ▶); cell refinement: *SAINT* (Bruker, 2009 ▶); data reduction: *SAINT*; program(s) used to solve structure: *SHELXTL* (Sheldrick, 2008 ▶); program(s) used to refine structure: *SHELXTL*; molecular graphics: *SHELXTL*; software used to prepare material for publication: *SHELXTL* and *PLATON* (Spek, 2009 ▶).

## Supplementary Material

Crystal structure: contains datablocks global, I. DOI: 10.1107/S1600536810000681/tk2611sup1.cif

Structure factors: contains datablocks I. DOI: 10.1107/S1600536810000681/tk2611Isup2.hkl

Additional supplementary materials:  crystallographic information; 3D view; checkCIF report

## Figures and Tables

**Table 1:** Hydrogen-bond geometry (Å, °)

*D*—H⋯*A*	*D*—H	H⋯*A*	*D*⋯*A*	*D*—H⋯*A*
O1—H1O1⋯O6^i^	0.86	1.69	2.5496 (13)	175
O1*W*—H1*W*1⋯O5^ii^	0.83	1.94	2.7585 (14)	167
O1*W*—H2*W*1⋯O6^iii^	0.90	1.94	2.8006 (15)	161
O3—H1O3⋯O5^ii^	0.78	2.14	2.7575 (19)	137
O4—H1O4⋯O1*W*^iv^	0.79	1.90	2.6784 (14)	170

Symmetry codes: (i) 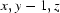; (ii) 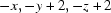; (iii) 
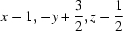
; (iv) 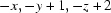.

**Fig. 1.**
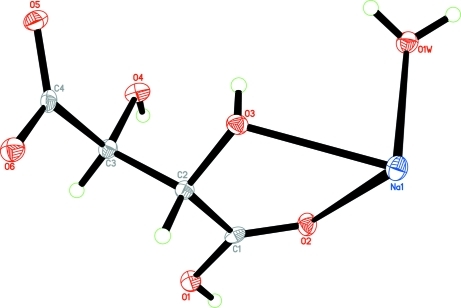
The asymmetric unit of the title compound, showing 50% probability displacement ellipsoids for non-H atoms and the atom-numbering scheme.

**Fig. 2.**
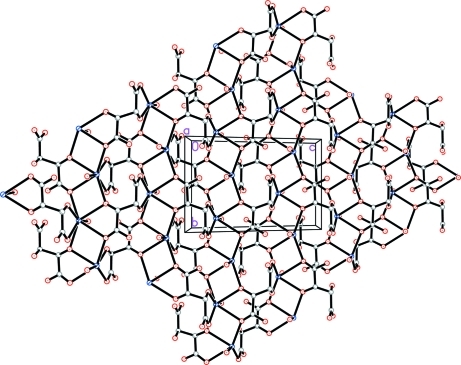
The polymeric structure of the title compound, viewed down the a axis, showing 2-dimensional array parallel to the (100) plane. All H atoms have been omitted for clarity.

**Fig. 3.**
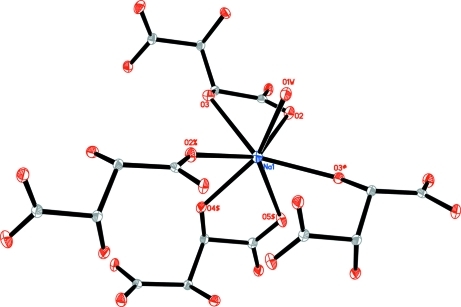
Part of a 2-dimensional array, highlighting the coordination environment for Na+. Displacement ellipsoids are drawn at the 50% probability level. H atoms have been omitted for clarity. Symmetry codes: (%) -x, 1/2 + y, 3/2 - z; (#) -x, -1/2 + y, 1/2 - z; ($) x, 3/2 - y, -1/2 + z.

**Fig. 4.**
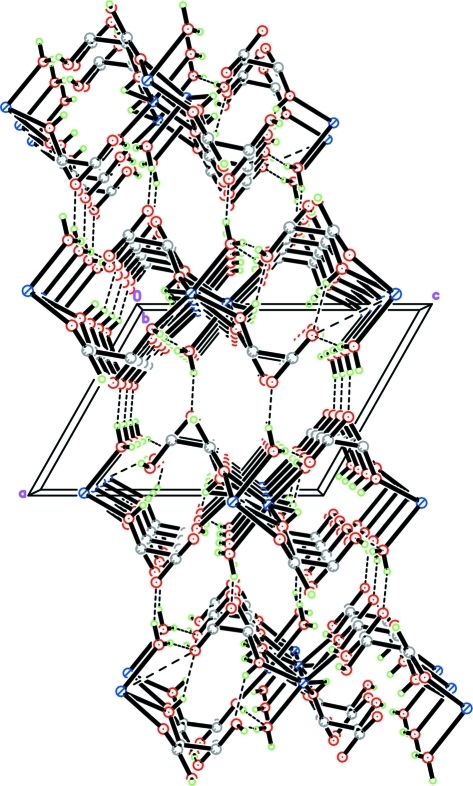
The crystal packing of the title compound, viewed down the b axis. Hydrogen bonds are shown as dashed lines. C-bound H atoms have been omitted for clarity.

## supplementary crystallographic information

### Comment

Tartaric acid is found free throughout nature, especially in many fruits and in wine, and as salts with Ca^2+^, K^+^, and Na^+^. It has many applications such as in making silver mirrors, in the manufacture of soft drinks, to provide tartness to foods, in tanning leather, and in making blueprints. Tartaric acid has optical activity (Synoradzki *et al.*, 2008).

Kubozono *et al.* (1993) reported the structure of the title compound, in the orthorhombic space group *P*2_1_2_1_2_1_. Herein, a new polymorph of the title compound is reported which crystallizes in the monoclinic space group *P*2_1_/*c*. The asymmetric unit contains a hydrogen tartrate anion, a Na cation and a water molecule (Fig. 1). The independent unit forms polymeric two-dimensional networks parallel to the plane (100) (Fig. 2). Each Na^+^ ion is surrounded by seven O atoms (Fig. 3) derived from a independent and three symmetry related hydrogen tartrate anions; and a water molecule, forming a distorted pentagonal bipyramidal geometry with Na—O distances ranging from 2.3331 (12) to 2.6740 (12) Å which are comparable to those reported in (Wong *et al.*, 2009), whereas the angles around the Na^+^ ion range from 62.55 (4) to 151.93 (4)°. Bond lengths and angles are within normal ranges and comparable to the orthorhombic polymorph of C_4_H_5_O_6_Na.H_2_O (Kubozono *et al.*, 1993).

The molecular structure is linked *via* intermolecular bifurcated O1W—H1W1···O5 and O3—H1O3···O5 acceptor bonds, generating *R*^1^_2_(6) ring motif (Bernstein *et al.*, 1995). In the crystal packing (Fig. 4), the polymeric two-dimensional arrays are linked by adjacent ring motifs, together with intermolecular O1—H1O1···O6, O1W—H2W1···O6 and O4—H1O4···O1W interactions, into a three-dimensional network.

### Experimental

Anhydrous tartaric acid (1.5 g, 0.1 mmol) was dissolved in water in a flat bottom flask with magnetic stirrer. In a separating funnel, sodium bicarbonate (0.85g, 0.1 mmol) was dissolved in water. The sodium bicarbonate solution was added in small portions to the flask of tartaric acid with stirring. The reaction mixture was refluxed for 1 h. After cooling the reaction mixture to room temperature, it was left for overnight stirring. The colourless crystals that subsequently formed were filtered and washed with methanol and dried at 353 K.

### Refinement

All H atoms were located in a difference Fourier map and fixed at these positions [C—H = 0.92–1.00 Å; O—H = 0.77–0.90 Å] and *U*_iso_(H) = 1.2 *U*_eq_(C) and 1.5 *U*_eq_(O).

### Crystal data

**Table:**

[Na(C_4_H_5_O_6_)(H_2_O)]	*F*(000) = 392
*M**_r_* = 190.09	*D*_x_ = 1.884 Mg m^−^^3^
Monoclinic, *P*2_1_/*c*	Mo *K*α radiation, λ = 0.71073 Å
Hall symbol: -P 2ybc	Cell parameters from 1671 reflections
*a* = 8.9723 (2) Å	θ = 2.6–29.9°
*b* = 7.1457 (1) Å	µ = 0.24 mm^−^^1^
*c* = 12.0186 (2) Å	*T* = 100 K
β = 119.571 (1)°	Needle, colourless
*V* = 670.18 (2) Å^3^	0.40 × 0.09 × 0.05 mm
*Z* = 4	

### Data collection

**Table:**

Bruker SMART APEXII CCD area-detector diffractometer	1947 independent reflections
Radiation source: fine-focus sealed tube	1532 reflections with *I* > 2σ(*I*)
graphite	*R*_int_ = 0.034
φ and ω scans	θ_max_ = 30.0°, θ_min_ = 2.6°
Absorption correction: multi-scan (*SADABS*; Bruker, 2009)	*h* = −12→12
*T*_min_ = 0.895, *T*_max_ = 0.979	*k* = −10→10
6649 measured reflections	*l* = −13→16

### Refinement

**Table:**

Refinement on *F*^2^	Primary atom site location: structure-invariant direct methods
Least-squares matrix: full	Secondary atom site location: difference Fourier map
*R*[*F*^2^ > 2σ(*F*^2^)] = 0.037	Hydrogen site location: inferred from neighbouring sites
*wR*(*F*^2^) = 0.092	All H-atom parameters refined
*S* = 1.03	*w* = 1/[σ^2^(*F*_o_^2^) + (0.0406*P*)^2^ + 0.2259*P*] where *P* = (*F*_o_^2^ + 2*F*_c_^2^)/3
1947 reflections	(Δ/σ)_max_ < 0.001
109 parameters	Δρ_max_ = 0.51 e Å^−^^3^
0 restraints	Δρ_min_ = −0.29 e Å^−^^3^

### Special details

**Table:**

Experimental. The crystal was placed in the cold stream of an Oxford Cyrosystems Cobra open-flow nitrogen cryostat (Cosier & Glazer, 1986) operating at 100.0 (1) K.
Geometry. All esds (except the esd in the dihedral angle between two l.s. planes) are estimated using the full covariance matrix. The cell esds are taken into account individually in the estimation of esds in distances, angles and torsion angles; correlations between esds in cell parameters are only used when they are defined by crystal symmetry. An approximate (isotropic) treatment of cell esds is used for estimating esds involving l.s. planes.
Refinement. Refinement of F^2^ against ALL reflections. The weighted R-factor wR and goodness of fit S are based on F^2^, conventional R-factors R are based on F, with F set to zero for negative F^2^. The threshold expression of F^2^ > 2sigma(F^2^) is used only for calculating R-factors(gt) etc. and is not relevant to the choice of reflections for refinement. R-factors based on F^2^ are statistically about twice as large as those based on F, and R- factors based on ALL data will be even larger.

### Fractional atomic coordinates and isotropic or equivalent isotropic displacement parameters (Å^2^)

**Table:**

	*x*	*y*	*z*	*U*_iso_*/*U*_eq_	
Na1	−0.01684 (8)	0.60710 (8)	0.68653 (6)	0.01242 (15)	
O1	0.40344 (13)	0.43067 (13)	1.07635 (10)	0.0119 (2)	
H1O1	0.4044	0.3103	1.0786	0.018*	
C1	0.26779 (18)	0.50448 (18)	0.97844 (15)	0.0099 (3)	
O1W	−0.25610 (13)	0.58582 (13)	0.72347 (11)	0.0129 (2)	
H1W1	−0.2558	0.6856	0.7595	0.019*	
H2W1	−0.3646	0.5522	0.6686	0.019*	
O2	0.15291 (13)	0.41821 (13)	0.89112 (11)	0.0130 (2)	
C2	0.26731 (18)	0.71881 (18)	0.98483 (14)	0.0088 (3)	
H2A	0.3650	0.7643	0.9739	0.011*	
O3	0.10842 (13)	0.79014 (13)	0.88733 (10)	0.0106 (2)	
H1O3	0.0405	0.7814	0.9094	0.016*	
C3	0.29897 (18)	0.78670 (18)	1.11559 (14)	0.0094 (3)	
H3A	0.4058	0.7442	1.1778	0.011*	
O4	0.16227 (13)	0.72503 (13)	1.13335 (11)	0.0128 (2)	
H1O4	0.1864	0.6265	1.1677	0.019*	
C4	0.30326 (18)	1.00147 (18)	1.11769 (14)	0.0100 (3)	
O5	0.19847 (14)	1.08865 (13)	1.13774 (11)	0.0148 (2)	
O6	0.41681 (13)	1.07517 (13)	1.09721 (11)	0.0122 (2)	

### Atomic displacement parameters (Å^2^)

**Table:**

	*U*^11^	*U*^22^	*U*^33^	*U*^12^	*U*^13^	*U*^23^
Na1	0.0142 (3)	0.0111 (3)	0.0123 (3)	−0.0020 (2)	0.0068 (3)	−0.0009 (2)
O1	0.0127 (5)	0.0075 (4)	0.0133 (6)	0.0015 (4)	0.0046 (5)	0.0013 (4)
C1	0.0125 (7)	0.0096 (6)	0.0112 (7)	0.0011 (5)	0.0087 (6)	0.0002 (5)
O1W	0.0130 (5)	0.0103 (5)	0.0156 (6)	−0.0014 (4)	0.0071 (5)	−0.0022 (4)
O2	0.0135 (5)	0.0099 (4)	0.0130 (6)	−0.0008 (4)	0.0045 (5)	−0.0016 (4)
C2	0.0092 (6)	0.0080 (6)	0.0085 (7)	0.0009 (5)	0.0040 (6)	0.0009 (5)
O3	0.0098 (5)	0.0119 (5)	0.0097 (5)	0.0016 (4)	0.0044 (4)	0.0012 (4)
C3	0.0107 (7)	0.0075 (6)	0.0100 (7)	0.0001 (5)	0.0050 (6)	0.0003 (5)
O4	0.0164 (5)	0.0093 (4)	0.0172 (6)	0.0014 (4)	0.0118 (5)	0.0030 (4)
C4	0.0107 (7)	0.0087 (6)	0.0071 (7)	−0.0006 (5)	0.0016 (6)	−0.0012 (5)
O5	0.0133 (5)	0.0108 (5)	0.0212 (6)	0.0009 (4)	0.0092 (5)	−0.0033 (4)
O6	0.0137 (5)	0.0092 (4)	0.0145 (6)	0.0000 (4)	0.0076 (5)	0.0006 (4)

### Geometric parameters (Å, °)

**Table:**

Na1—O4^i^	2.3331 (12)	C2—O3	1.4201 (17)
Na1—O1W	2.4020 (12)	C2—C3	1.531 (2)
Na1—O3^ii^	2.4235 (11)	C2—H2A	1.0020
Na1—O3	2.4741 (12)	O3—Na1^iii^	2.4235 (11)
Na1—O2^iii^	2.4858 (11)	O3—H1O3	0.7774
Na1—O2	2.5479 (12)	C3—O4	1.4150 (16)
Na1—O5^i^	2.6740 (12)	C3—C4	1.5350 (18)
O1—C1	1.3155 (18)	C3—H3A	0.9288
O1—H1O1	0.8604	O4—Na1^iv^	2.3332 (11)
C1—O2	1.2149 (18)	O4—H1O4	0.7905
C1—C2	1.5336 (18)	C4—O5	1.2465 (17)
O1W—H1W1	0.8339	C4—O6	1.2741 (17)
O1W—H2W1	0.8983	O5—Na1^iv^	2.6740 (12)
O2—Na1^ii^	2.4858 (11)		
			
O4^i^—Na1—O1W	151.93 (4)	C1—O2—Na1^ii^	145.91 (10)
O4^i^—Na1—O3^ii^	122.35 (4)	C1—O2—Na1	114.40 (9)
O1W—Na1—O3^ii^	80.57 (4)	Na1^ii^—O2—Na1	99.35 (4)
O4^i^—Na1—O3	87.29 (4)	O3—C2—C3	109.60 (11)
O1W—Na1—O3	82.55 (4)	O3—C2—C1	110.09 (11)
O3^ii^—Na1—O3	139.47 (4)	C3—C2—C1	111.35 (11)
O4^i^—Na1—O2^iii^	73.41 (4)	O3—C2—H2A	111.2
O1W—Na1—O2^iii^	78.92 (4)	C3—C2—H2A	107.5
O3^ii^—Na1—O2^iii^	133.11 (4)	C1—C2—H2A	107.0
O3—Na1—O2^iii^	78.23 (4)	C2—O3—Na1^iii^	131.19 (8)
O4^i^—Na1—O2	111.86 (4)	C2—O3—Na1	113.77 (8)
O1W—Na1—O2	87.20 (4)	Na1^iii^—O3—Na1	103.19 (4)
O3^ii^—Na1—O2	77.97 (4)	C2—O3—H1O3	109.0
O3—Na1—O2	64.61 (4)	Na1^iii^—O3—H1O3	91.1
O2^iii^—Na1—O2	141.72 (4)	Na1—O3—H1O3	103.6
O4^i^—Na1—O5^i^	62.55 (4)	O4—C3—C2	108.69 (11)
O1W—Na1—O5^i^	144.66 (4)	O4—C3—C4	109.00 (11)
O3^ii^—Na1—O5^i^	65.28 (3)	C2—C3—C4	108.91 (12)
O3—Na1—O5^i^	117.44 (4)	O4—C3—H3A	113.8
O2^iii^—Na1—O5^i^	131.27 (4)	C2—C3—H3A	108.5
O2—Na1—O5^i^	77.41 (4)	C4—C3—H3A	107.8
C1—O1—H1O1	114.8	C3—O4—Na1^iv^	130.28 (8)
O2—C1—O1	125.82 (12)	C3—O4—H1O4	108.8
O2—C1—C2	121.86 (13)	Na1^iv^—O4—H1O4	111.3
O1—C1—C2	112.32 (12)	O5—C4—O6	125.60 (12)
Na1—O1W—H1W1	105.6	O5—C4—C3	119.18 (12)
Na1—O1W—H2W1	128.7	O6—C4—C3	115.22 (12)
H1W1—O1W—H2W1	109.5	C4—O5—Na1^iv^	118.45 (9)
			
O1—C1—O2—Na1^ii^	13.8 (3)	O4^i^—Na1—O3—C2	−82.64 (9)
C2—C1—O2—Na1^ii^	−165.96 (11)	O1W—Na1—O3—C2	123.57 (9)
O1—C1—O2—Na1	−157.55 (11)	O3^ii^—Na1—O3—C2	57.61 (8)
C2—C1—O2—Na1	22.65 (16)	O2^iii^—Na1—O3—C2	−156.25 (9)
O4^i^—Na1—O2—C1	46.35 (11)	O2—Na1—O3—C2	33.14 (8)
O1W—Na1—O2—C1	−112.44 (10)	O5^i^—Na1—O3—C2	−25.64 (10)
O3^ii^—Na1—O2—C1	166.61 (10)	O3—C2—C3—O4	58.74 (14)
O3—Na1—O2—C1	−29.37 (9)	C1—C2—C3—O4	−63.32 (14)
O2^iii^—Na1—O2—C1	−44.32 (10)	O3—C2—C3—C4	−59.89 (14)
O5^i^—Na1—O2—C1	99.59 (10)	C1—C2—C3—C4	178.05 (11)
O2—C1—C2—O3	7.77 (19)	C2—C3—O4—Na1^iv^	−126.79 (10)
O1—C1—C2—O3	−172.06 (11)	C4—C3—O4—Na1^iv^	−8.22 (17)
O2—C1—C2—C3	129.54 (15)	O4—C3—C4—O5	2.38 (19)
O1—C1—C2—C3	−50.29 (15)	C2—C3—C4—O5	120.82 (15)
C3—C2—O3—Na1^iii^	66.43 (14)	O4—C3—C4—O6	−177.16 (12)
C1—C2—O3—Na1^iii^	−170.76 (8)	C2—C3—C4—O6	−58.73 (17)
C3—C2—O3—Na1	−157.80 (8)	O6—C4—O5—Na1^iv^	−177.69 (12)
C1—C2—O3—Na1	−34.99 (13)	C3—C4—O5—Na1^iv^	2.82 (18)

Symmetry codes: (i) *x*, −*y*+3/2, *z*−1/2; (ii) −*x*, *y*−1/2, −*z*+3/2; (iii) −*x*, *y*+1/2, −*z*+3/2; (iv) *x*, −*y*+3/2, *z*+1/2.

### Hydrogen-bond geometry (Å, °)

**Table:**

*D*—H···*A*	*D*—H	H···*A*	*D*···*A*	*D*—H···*A*
O1—H1O1···O6^v^	0.86	1.69	2.5496 (13)	175
O1W—H1W1···O5^vi^	0.83	1.94	2.7585 (14)	167
O1W—H2W1···O6^vii^	0.90	1.94	2.8006 (15)	161
O3—H1O3···O5^vi^	0.78	2.14	2.7575 (19)	137
O4—H1O4···O1W^viii^	0.79	1.90	2.6784 (14)	170

Symmetry codes: (v) *x*, *y*−1, *z*; (vi) −*x*, −*y*+2, −*z*+2; (vii) *x*−1, −*y*+3/2, *z*−1/2; (viii) −*x*, −*y*+1, −*z*+2.
